# A Systematic Analysis of the 3′UTR of *HNF4A* mRNA Reveals an Interplay of Regulatory Elements Including miRNA Target Sites

**DOI:** 10.1371/journal.pone.0027438

**Published:** 2011-11-30

**Authors:** Andrea Wirsing, Sabine Senkel, Ludger Klein-Hitpass, Gerhart U. Ryffel

**Affiliations:** Institut für Zellbiologie (Tumorforschung), Universitätsklinikum Essen, Universität Duisburg-Essen, Essen, Germany; UMDNJ-New Jersey Medical School, United States of America

## Abstract

Dysfunction of hepatocyte nuclear factor 4α (HNF4α) has been linked to maturity onset diabetes of the young (MODY1), diabetes type II and possibly to renal cell carcinoma (RCC). Whereas diabetes causing mutations are well known, there are no *HNF4A* mutations found in RCC. Since so far analyses have been constricted to the promoter and open reading frame of *HNF4A*, we performed a systematic analysis of the human *HNF4A* 3′UTR. We identified a short (1724 nt) and long (3180 nt) 3′UTR that are much longer than the open reading frame and conferred a repressive effect in luciferase reporter assays in HEK293 and INS-1 cells. By dissecting the 3′UTR into several pieces, we located two distinct elements of about 400 nt conferring a highly repressive effect. These negative elements A and B are counteracted by a balancer element of 39 nt located within the 5′ end of the *HNF4A* 3′UTR. Dicer knock-down experiments implied that the *HNF4A* 3′UTR is regulated by miRNAs. More detailed analysis showed that *miR-34a* and *miR-21* both overexpressed in RCC cooperate in downregulation of the *HNF4A* mRNA. One of the identified miR-34a binding sites is destroyed by SNP rs11574744. The identification of several regulatory elements within the *HNF4A* 3′UTR justifies the analysis of the 3′UTR sequence to explore the dysfunction of HNF4α in diabetes and RCC.

## Introduction

Hepatocyte nuclear factor 4α (HNF4α) is a highly conserved transcription factor that is predominantly expressed in liver, kidney, pancreas and intestine [1], [2]. The impact of HNF4α on gene regulation has been elucidated by identifying hundreds of functional target genes involved in various processes such as homeostasis, metabolism, immune and stress response, cell structure, apoptosis and cancer [3]–[6]. Mutations in the *HNF4A* gene are linked to diabetes type II [7], [8] and maturity onset diabetes of the young type 1 (MODY1) [9], [10]. Furthermore, several data indicate that HNF4α might act as a tumor suppressor whose inactivation leads to carcinogenesis. Thus, re-expression of HNF4α in murine hepatocellular carcinoma (HCC) retarded tumor growth of subcutaneous transplanted cells [11], [12]. In addition, human renal cell carcinomas (RCC) show a 4.7 fold downregulation in *HNF4A* mRNA level [13] and the abundance as well as DNA binding activity of its protein is frequently reduced in tumors compared to normal tissue [14]. The tumor repressive effect is supported by findings that HNF4α inhibits cell proliferation in various cell types, including murine hepatocellular carcinoma cells [11], [12], endothelial lung and embryonal carcinoma cells [15], insulinoma cells [4] as well as embryonic kidney cells [5], [16].

The transcriptional regulation of *HNF4A* is quite well understood and involves two promoters, P1 and P2, which mediate cell specific activity [17]–[19]. The importance of the P2 promoter in β-cells of the pancreas is revealed by five distinct mutations that occur in various promoter elements and are linked to maturity onset diabetes of the young 1 (MODY1) [20]. These mutations in regulatory sequences of *HNF4A* complement the numerous MODY1 mutations found in the open reading frame (ORF) [21]. However, so far no mutation in the *HNF4A* gene has been identified that may explain the downregulation of HNF4α in RCC [22], [23]. Clearly, regulation via the 3′ untranslated region (3′UTR) of the mRNA is a possible option. In fact, the 3′UTR of the *HNF4A* mRNA based on RefSeq NM_000457.3 is about 1.7 kb in length and thus longer than the ORF with its 1.4 kb. So far the function of the 3′UTR has not been analyzed and this lack of knowledge is typical for most other mRNAs as well. It reflects the limited insight into functional elements in the 3′UTR although they are known to play an important role in translation, localization as well as stability of mRNAs [24], [25]. Whereas the interaction of the 3′UTR with specific RNA binding proteins has been known for a long time [26], [27], the binding of microRNA (miRNA) as an important regulatory event has been recognized more recently [28], [29].

miRNAs are expressed in a cell-specific manner and have been implicated in the posttranscriptional regulation of target mRNAs resulting in decreased protein expression [28], [29]. By modulating oncogenic and tumor suppessor pathways, miRNAs have been shown to contribute to tumorigenesis [30]–[32]. miRNA expression profiling in RCC has revealed a large number of miRNAs that are either up- or downregulated in the tumors compared to normal tissue [33]–[39]. Whether these misregulated miRNAs affect *HNF4A* expression is not known. miRNAs also play a role in the developing pancreas including the β-cells of the Langerhans islands [40], [41]. However, it is not known, whether they are dysregulated in diabetes type II or MODY and affect mRNAs such as *HNF4A* whose dysfunction leads to impaired insulin secretion.

In the present study we elucidate the regulatory potential of the 3′UTR of the *HNF4A* mRNA to expose the possible regulation of *HNF4A* in RCC, MODY1 and diabetes type II. We performed a systematic analysis of the entire *HNF4A* 3′UTR to reveal distinct regulatory elements controlling HNF4α expression.

## Materials and Methods

### Plasmid construction

All primers are listed in Table S1. The *HNF4A* 3′UTR was amplified using a human BAC clone (RPCIB753B08466Q; imaGenes) as a template and primers containing flanking *Spe*I or *Xba*I and *Not*I sites. The cleaved *HNF4A* 3′UTR and all shortened fragments were cloned into *Xba*I/*Not*I sites of the *Renilla* luciferase reporter pRL-Con [42] and subsequently sequenced.

The sequence 631–3180 of the corresponding construct was excised from construct 1–3180 with *Xba*I/*Not*I and ligated into the same sites of the RL-Con plasmid. To delete the sequence containing negative element A and B and obtain constructs 1–844+1720–3180 and 631–844+1720–3180, we used two *Eco*RI sites present in the *HNF4A* 3′UTR sequence. The latter construct was also used to generate construct 631–849+1718–850+1719–3180, by re-introducing the excised *Eco*RI-fragment and selecting for clones containing this sequence in 3′-5′ direction. To get constructs 1–630+850–1207 and 1–630+1288–1666, we cleaved the construct containing the 5′ 843 nt of the *HNF4A* 3′UTR with *Xba*I/*Not*I and inserted the *Xba*I/*Not*I cleaved PCR products from 850–1207 and 1288–1666, respectively. To determine if negative element A and B function on RNA level, we introduced a *Xba*I/*Spe*I cleaved PCR product containing the SV40 PAS into the *Xba*I site upstream of negative element A and B constructs. Both orientations of the insert were identified by sequencing, resulting in construct 5′-3′ PAS + 850–1207, 3′-5′ PAS + 850–1207, 5′-3′ PAS + 1288–1666 and 3′-5′ PAS + 1288–1666. To locate the balancer, PCR fragments with flanking *Spe*I and *Xba*I sites were introduced in both orientations into the *Xba*I site of the construct containing negative element A. For mutation analysis the balancer oligonucleotides listed in Table S2 were inserted into the 850–1207 construct whose *Xba*I site was replaced with the polylinker oligonucleotide. For insertion of the mut2 mutation into the long 3′UTR construct 1–3180, the *Apa*I/*Xba*I fragment of the 3′UTR (137–632) was cloned into pBluescript II SK+ and mutated with the QuikChange II Site-Directed Mutagenesis Kit (Stratagene). The sequence verified fragment was reinserted into the wild type construct. The site directed mutation in the proximal miR-34a site was made with the same mutagenesis kit. The most relevant DNA constructs have been deposited at addgene.

### Cell culture

Dicer-kd/2b2 cells [42] were grown in DMEM (Gibco-BRL) supplemented with 10% heat-inactivated fetal calf serum (FCS), penicillin/streptomycin (100 U/ml), 2 mM glutamine, 10 µg/ml blasticidin and 250 µg/ml zeocin (Invitrogen). The INS-1#5.3-19 cell line [43] was cultured in RPMI-1640 medium supplemented with 10% heat inactivated FCS, penicillin/streptomycin (100 U/ml), 1 mM sodium pyruvate, 10 mM HEPES, 2 mM glutamine, 50 µM mercaptoethanol, 10 µg/ml blasticidin and 200 µg/ml zeocin. HepG2 cells [22] were maintained in DMEM (Gibco-BRL) supplemented with 10% heat-inactivated fetal calf serum (FCS), penicillin/streptomycin (100 U/ml) and 2 mM glutamine. The human kidney cell line HK120 was kindly provided by Stilla Frede (Institut für Physiologie, Universität Duisburg-Essen, Essen, Germany) and grown in RPMI-1640 medium supplemented with 10% heat inactivated FCS, penicillin/streptomycin (100 U/ml) and 2 mM glutamine.

### Transient transfection and luciferase assay

For each assay cells were seeded into a 96-well plate 24 h before transfection. Cells were transiently transfected with 40 ng of DNA comprised of Rc/CMV (Invitrogen), of 0.01 ng firefly luciferase construct CMV-luc (Rc/CMV derivative containing the firefly luciferase) for normalization of transfection efficiencies and of the *Renilla* luciferase reporter constructs in pRL-Con [42] using 0.05–0.08 ng to obtain equal molar amounts for the constructs with various length inserts. 24 h after transfection using FuGeneHD (Roche) *Renilla* and firefly luciferase activities were measured with the Dual-Luciferase Reporter Assay Kit (Promega). Normalized *Renilla* activity in cells transfected with pRL-Con was set to 100% and used for standardization.

For Dicer knock-down experiments, expression of the short hairpin targeting Dicer [42] was induced with 1 µg/ml of doxycycline for three or seven days. Transfection assays were performed as described above by using 0.08 ng of RL reporter plasmids. Four hours later addition of doxycycline was repeated and 48 h after transfection, luciferase activities were determined. The normalized values for each construct obtained for uninduced cells, which were treated with ethanol, was set to 100% and used for standardization.

To determine the impact of miRNAs on the *HNF4A* 3′UTR, we co-transfected 0.08 ng of the pRL-Con reporter plasmids with 0.01 ng of the CMV-luc construct and 50 ng of pcDNA3.1 *pri-miR-34a* [44] or pCMV-*mir-21* [45] using either Rc/CMV (Invitrogen) or pcDNA3.1 (Invitrogen) as a negative control. Luciferase activities were determined 24 h after transfection as described above.

### 3 ′RACE and qRT-PCR

Total RNA was isolated from HepG2 and HK120 cells using peqGold RNAPure (PeqLab) according to the manufacturer's instruction. cDNA was synthesized using the High Capacity cDNA Reverse Transcription Kit (Applied Biosystems) together with an oligo-dT-adapter primer (5′ GGCCACGCGTCGACTAGTACTTTTTTTTTTTTTTTTT 3′). PCR was performed (FailSafe PCR System, EPICENTRE) using a sense gene specific primer (proximal PA: 5′ CGGGATCCGGCTGCACTAAAATTCACTTAGGGTCG 3′; distal PA: 5′ CGGGATCCTTCTTACTCTTCTGTGTTTTAACAAAA 3′) and an antisense adapter primer (5′ CCACGCGTCGACTAGTACTTT 3′). PCR products were analyzed on an agarose gel and either cloned into pBluescript and sequenced or sequenced directly.

SYBR-Green real time PCR was performed on a 7900HT Sequence Detection System (Applied Biosystems) using Power-SYBR Green Mix (Applied Biosystems). Templates were determined in triplicate and the housekeeping gene *GAPDH* served as a reference. To check for DNA contamination, control reactions without reverse transcriptase were performed. The primers used were the same as described above.

### miRNA expression profile

RNA was isolated from HEK293 cells [16] by using the mirVana™ RNA isolation kit (Ambion) according to the manufacturer's instruction. RNA samples (20 ng) were reverse transcribed (384 TaqMan miRNA assay, beta version, Applied Biosystems) using eight different 48plex stem-loop RT primer pools. The cDNAs were quantified by real-time PCR using the corresponding 8×48 individual miRNA Taqman Assays in duplicate reactions (10 µl) containing 0.1 ng of cDNA, 1× Universal Master Mix and 1× assay. Data were analyzed by the ΔCT method using RNU48 as a normalization control.

### *In silico* analyses

Target sites for 20 miRNAs (Table S3) were predicted within the 3180 nt *HNF4A* 3′UTR with RNA22 [46] using 1, 7, 14 and −20 for unpaired bases, seed/nucleus in nucleotides, minimum number of paired-up bases and maximum folding energy in heteroduplex, respectively. The proximal miR-34a target site in the 5′ 449 nt of the *HNF4A* 3′UTR was predicted with TargetScan (http://targetscan.org/). The UTRdb database [47] was used to identify regulatory motifs within the *HNF4A* 3′UTR. MIRb and MIRc were predicted using the RepeatMasker function from the UCSC Genome Browser of Human Feb. 2009 Assembly (http://www.genome.ucsc.edu/cgi-bin/hgGateway).

## Results

### Two alternative 3′UTRs of the human *HNF4A* mRNA

**Figure 1:**
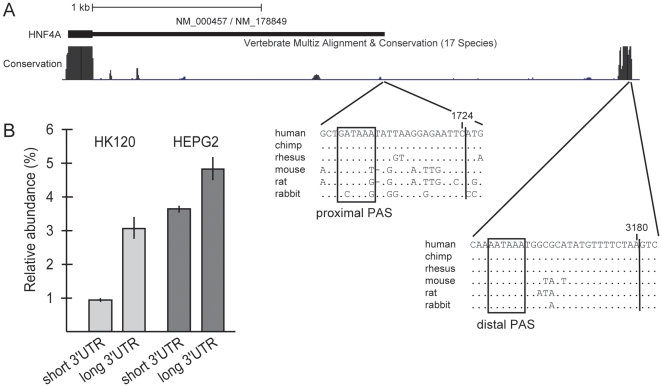
Two distinct polyadenylation signals (PAS) in the human *HNF4A* mRNA. (**A**) Schematic representation of the *HNF4A* 3′UTR. The screen shot taken from the UCSC Genome Browser (assembly March 2006) depicts the known human *HNF4A* 3′UTR with the RefSeq sequences NM_000457.3 and NM_178849.1 and the genome position from 42,491,540 to 42,494,950 of chromosome 20. The degree of conservation across 17 species is indicated by black areas. The nucleotide sequence alignment of the region surrounding the proximal and distal PAS is shown below. Non-conserved nucleotides in comparison to the human sequence are given for the different species, while dots represent conserved nucleotides. The proximal and distal PAS are boxed and the corresponding cleavage sites, as determined by 3′ RACE and subsequent sequencing, are indicated by a vertical line. The last nucleotide of the short and long 3′UTR is marked at position 1724 and 3180, respectively. (**B**) The relative abundance of the short and long *HNF4A* 3′UTRs was determined in comparison to the house keeping gene *GAPDH*. Two independent RNA samples were prepared from each cell line and the qRT-PCR was performed in triplicates. Each column thus represents the mean±SD of six measurements.

The RefSeq sequences NM_000457.3 and NM_178849.1 of the human *HNF4A* mRNA encode a 3′UTR of 1724 nt that contains the non-canonical polyadenylation signal (PAS) GATAAA [48] 15 nt upstream of the 3′ end (Fig. 1A). However, this PAS and the surrounding sequences are conserved in primates only, but not among other mammals. In contrast, the 3′UTR of murine *Hnf4a* mRNA is 2816 nt in length (RefSeq NM_008261.2) and encompasses the canonical PAS [48] AATAAA (Fig. 1A). This PAS and the surrounding sequences are highly conserved among different mammals including human. To determine which polyadenylation site is functional in human cells, we performed 3′ RACE with *HNF4A* mRNA isolated from the hepatoblastoma cell line HepG2 and the kidney cell line HK120. Sequence analyses of the cDNA revealed that in both cell lines the proximal as well as the distal PAS are used resulting in cleavage of the mRNA 15 nt and 19 nt downstream of the PAS at position 1724 nt and 3180 nt, respectively.

To determine the abundance of the short (1–1724) and long (1–3180) 3′UTR in the human *HNF4A* RNA of the HepG2 and HK120 cell lines, we performed qRT-PCR using *GAPDH* as a reference. The amount of the *HNF4A* 3′UTR was about two-fold higher in HepG2 than in HK120 cells (Fig. 1B). In both cell lines the distal PAS generating the long 3′UTR (1–3180) was used frequently, representing about 75% and 60% of the *HNF4A* transcripts in the hepatoblastoma and kidney cell line, respectively. These data reveal that in human cells in addition to the predicted *HNF4A* 3′UTR of 1724 nt, a much longer 3′UTR of 3180 nt is expressed predominantly.

### Repressive effect of the *HNF4A* 3′UTR

**Figure 2:**
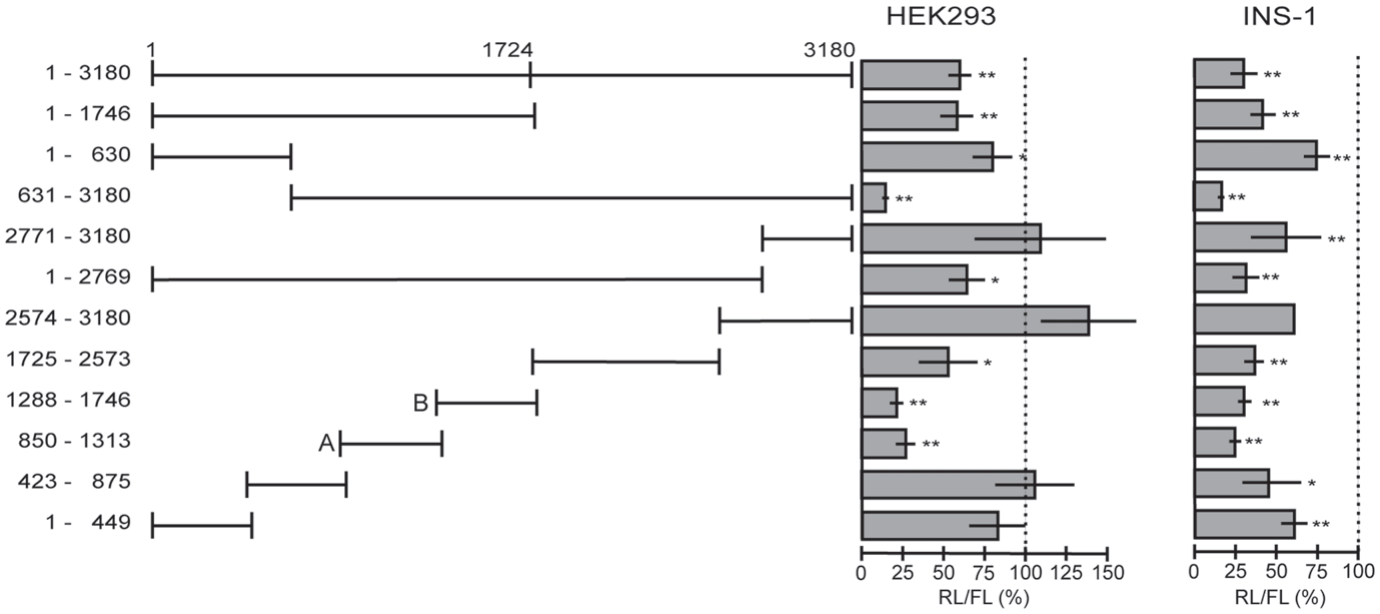
Systematic reporter analyses of the human *HNF4A* 3′UTR. The results of luciferase assays 24 h after transient transfection into HEK293 and INS-1 cells are shown. The numbers of the construct names refer to the nucleotide position in the *HNF4A* 3′UTR with 1 being the first nucleotide after the stop codon. Each 3′UTR fragment was cloned downstream of the *Renilla* luciferase ORF into the RL-Con plasmid. At least three transfection assays were performed for each construct, involving two independent plasmid preparations. Each assay was performed in triplicate and as indicated by RL/FL a CMV-driven firefly luciferase (FL) was used to control for transfection efficiency. The activity of the empty RL-Con plasmid was used for standardization to 100%. *p*-values were determined using a one-sample *t* test. *p*-values of <0.05 and of <0.01 are indicated by * or **, respectively.

To gain insight into the mode of regulation of *HNF4A* via its 3′UTR, we performed an *in silico* search for regulatory elements. Since we found only few potential *cis*-elements of RNA binding proteins, but several hundred possible target sites for miRNAs, we decided to systematically use a set of functional assays to locate regulatory elements. We cloned the short (1–1746) and long (1–3180) 3′UTR downstream of the *Renilla* luciferase ORF into the reporter plasmid RL-Con [42] and analyzed the effect in human embryonic kidney (HEK293) cells using a firefly luciferase reporter as reference (Fig. 2, middle panel). The *Renilla* luciferase activity was significantly reduced to about 60% by insertion of the long (1–3180) or short (1–1746) 3′UTR implying the existence of elements conferring a repressive effect.

To locate these negative acting elements, we dissected the 3′UTR and located a repressive activity in the 5′ part (1–630), whereas the 3′ part (2771–3180) had no influence. Furthermore, we observed a distinct activity located in fragment 631–3180 that surprisingly had a much higher repressive effect, while a construct containing the sequence from 1–2769 resulted in a decrease in luciferase activity similar to the one observed for the long 3′UTR. To narrow down the area within the *HNF4A* 3′UTR which confers the strong repressive effect, we generated short, mainly overlapping constructs covering the entire 3180 nt of the 3′UTR. Whereas the majority of 3′UTR fragments showed no or only minor effects, the two constructs comprising the 3′UTR sequences A (850–1313) and B (1288–1746) repressed luciferase activity down to 27% and 21%, respectively. Since in HEK293 cells the *HNF4A* gene is silent [16], we measured the activity of the same 3′UTR fragments in the rat insulinoma cell line INS-1 expressing *Hnf4a* [43]. In this cell line a similar pattern of repression of the individual constructs was observed, but the effect was even more pronounced (Fig. 2, right panel). Taken together the *HNF4A* 3′UTR contains several elements that negatively influence luciferase reporter activity.

### Identification of negative element A and B within the *HNF4A* 3′UTR

Using UCSC Genome Browser we identified the “mammalian interspersed repetitive elements” MIRb and MIRc within sequence A (850–1313) and B (1288–1746), respectively (Fig. S1). However, we excluded that these repetitive elements mediate the repressive effects, as fragments retaining the sequence for MIRb (1208–1313) or MIRc (1392–1513) did not affect luciferase reporter activity in HEK293 and INS-1 cells (Fig. S1, middle and right panels).

To locate the functional sequences we gradually trimmed the sequences from the 3′ and 5′ end (Fig. S1, left panel). Shortening sequence A on the 3′ end to position 1259 and even to 1207 amplified the repressive effect in HEK293 cells to 16% and 15%, respectively (Fig. S1, middle panel). Further constriction on either side revealed a gradual release of the repressive effect. Therefore, we defined the fragment extending from 850–1207 as negative element A. Similarly, shortening sequence B on the 3′ and 5′ end, we defined negative element B (1288–1666), as it mediates the highest repressive function and any truncation leads to a partial or even total loss of the repressor activity (Fig. S1, left panel). A corresponding analysis in INS-1 cells gave a most similar result (Fig. S1, right panel).

Taken together we located two previously unknown negative elements within the *HNF4A* 3′UTR that are separated by about 80 nt. Their size of approximately 400 nt (357 nt and 378 nt) is quite large and both are present in the short (1–1724) as well as the long (1–3180) 3′UTR of the *HNF4A* mRNA.

### A balancer counteracting the negative elements

**Figure 3:**
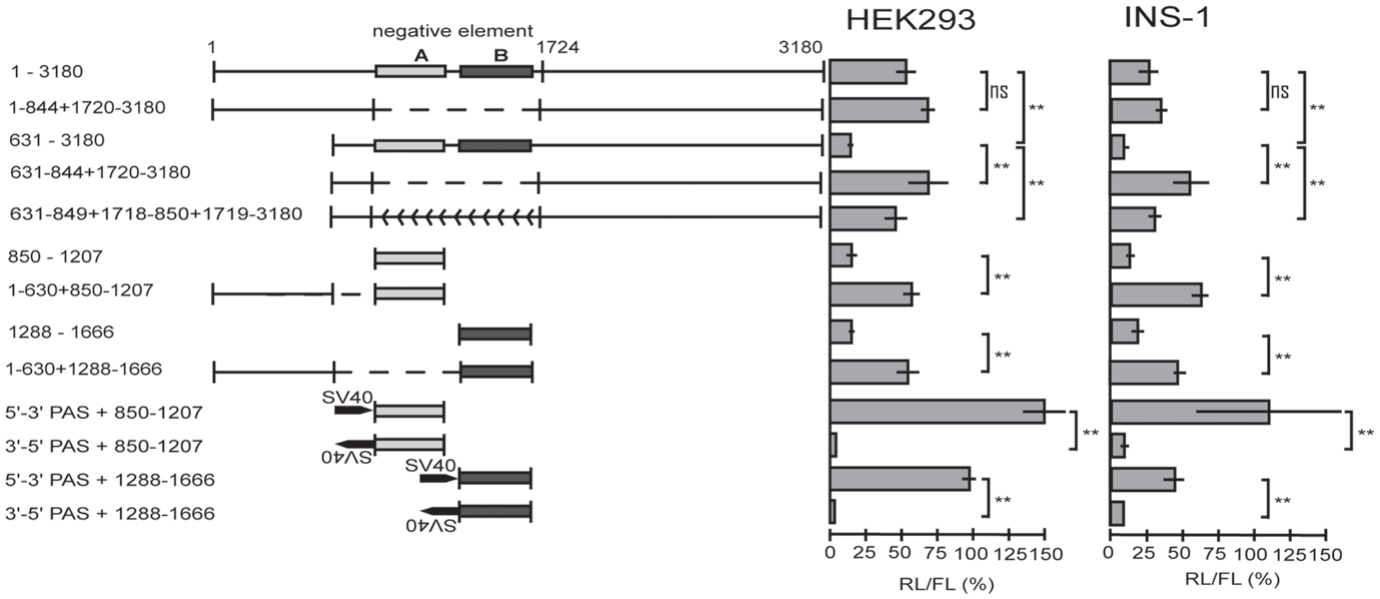
Balancer counteracting the negative elements A and B. The negative elements A and B are indicated by light grey and dark grey boxes, respectively. The deletion of the negative elements is illustrated by a broken line, whereas the inversion of this element is marked by backwards arrows. The insertion of the SV40 termination signal in sense and antisense is marked. The results of luciferase assays were derived and evaluated as in Fig. 2. *p*-values were determined between two columns as indicated by brackets using an independent-samples *t* test. Non-significant changes are marked by ns and refer to *p*-values>0.05 and ** refers to p-values<0.01.

The strong activity of negative element A and B was only observed when these elements were excised from the 3′UTR (Fig. 2). Deleting a sequence containing both negative elements from the construct containing the long (1–3180) *HNF4A* 3′UTR did not change the luciferase activity in comparison to the entire 3′UTR (Fig. 3). In contrast, the high repressive effect of construct 631–3180, was abolished upon deletion or inversion of the sequence containing negative element A and B (Fig. 3). This implied a counteracting element in the 5′ part of the 3′UTR (1–630) we refer to as a balancer. Indeed, insertion of the sequence 1–630 nt upstream of element A or B largely abolished the repressive effect of the negative element A or B (Fig. 3). The observation that the repressive effect of element A and B is lost upon inversion is consistent with a regulatory element functioning on the RNA level. To support this notion, we inserted the SV40 3′UTR with its PAS upstream of negative element A or B. In both cases the repressive effect was lost, as expected if the transcript is polyadenylated at the SV40 PAS and thus does not include negative element A or B (Fig. 3). However, the abolishment of the repressive effect was not seen, if the SV40 3′UTR was inserted in opposite direction leading to a PAS on the non-coding DNA (Fig. 3). Additionally, this experiment excluded the possibility that any sequence introduced upstream of element A or B would abrogate the effect. All described effects were highly similar in HEK293 and INS-1 cells.

**Figure 4:**
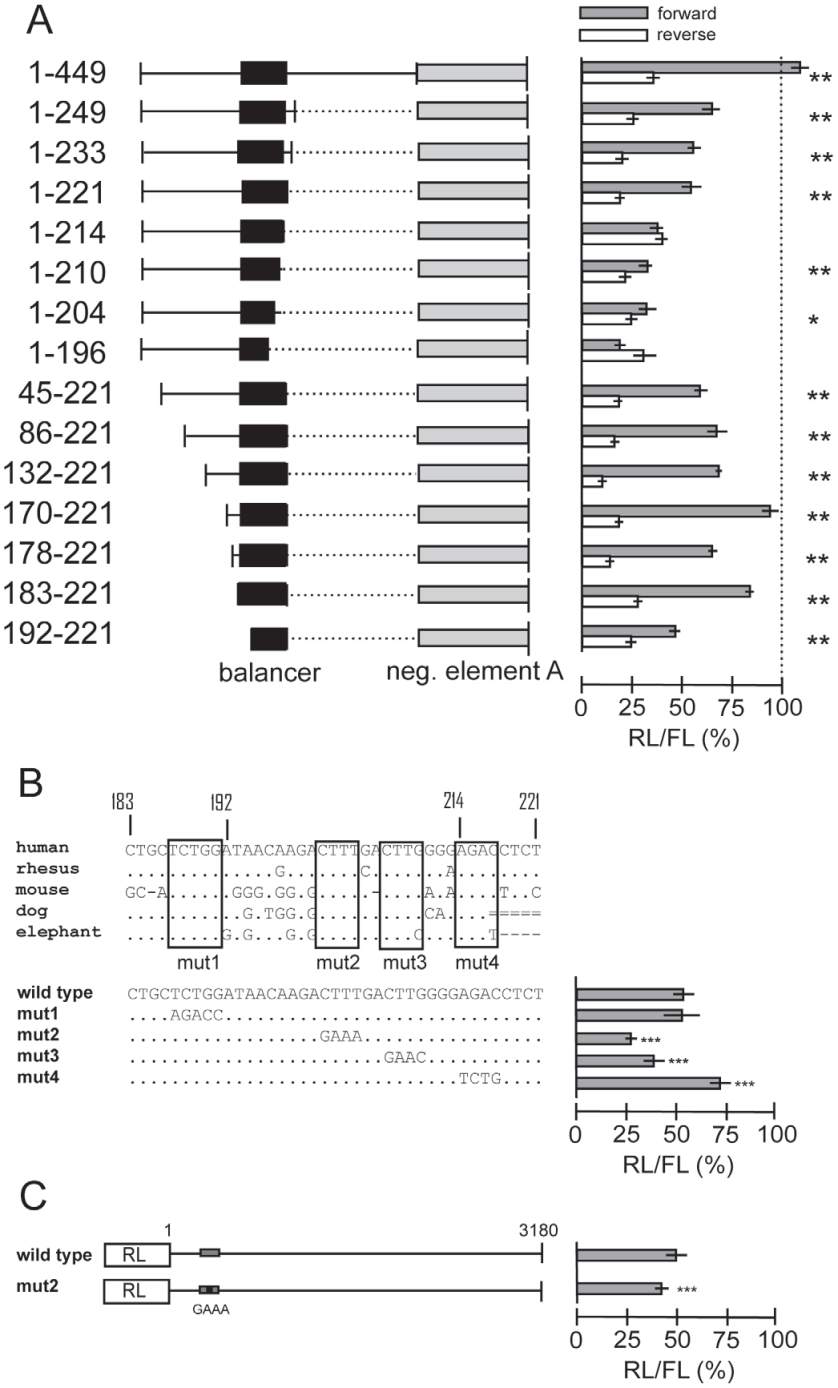
Mapping of the balancer element counteracting the negative elements. (**A**) The indicated 5′ sequences of the *HNF4A* 3′UTR were cloned upstream of negative element A (850–1207, light grey box). The identified balancer is marked by a black box. Luciferase assays were performed in INS-1 cells as described for Fig. 2. Grey and white bars represent results obtained with the constructs containing the 5′ sequences in front of negative element A in forward (5′-3′) and reverse orientation (3′-5′), respectively. The results of luciferase assays were derived and evaluated as in Fig. 2. *p*-values of<0.01 and of <0.001, determined between the forward and reverse orientation of each construct, are indicated by * or **, respectively. (**B**) The balancer sequence identified in the human 3′UTR of *HNF4A* was aligned to the corresponding sequence of other mammals using the UCSC Genome Browser (hg18, multiz alignments of 44 vertebrates). The four regions conserved are boxed. Below, the wild type balancer sequence and the four mutants tested in front of the negative element A (850–1207) are given. Their performance in the luciferase assay in INS-1 cells is summarized from at least three independent preparation of each construct. *p*-values of<10^−5^ (***) are calculated in comparison to the wild type sequence using an independent-samples *t* test. (**C**) The long (1–3180) 3′UTR linked downstream of the *Renilla* luciferase (RL) and a corresponding construct containing mut2 given in panel B were assayed in INS-1 cells. The balancer (grey box) and mut2 (black box) are not drawn to scale. Eight and ten independent plasmid preparations were used for the wild type and mut2 construct, respectively. *p*-values of<10^−5^ (***) are calculated in comparison to the wild type sequence using an independent-samples *t* test.

To define the sequence requirements of the balancer we further truncated the 3′UTR fragment 1–630 on both sides and measured its activity to counteract the negative element A (Fig. 4A). As inversion of the balancer sequence abolished its function (see construct 1–449 in Fig. 4A), we evaluated the effect of the balancer in each deletion construct by comparing the activity in forward and reverse orientation. Using this criterion we defined the sequence from 183–221, i.e. 39 nt, as the minimal balancer, as a more than twofold difference between the forward and reverse construct was measured. Since the balancer sequence is partially conserved in mammals (Fig. 4B), we introduced four mutations targeting the conserved sequence blocks and evaluated their effects. Clearly, the mutations mut2 and mut3 impair the balancer function indicating that the conserved CTTT and CTTG blocks are crucial elements. The observation that the mutation mut1 does not interfere with the function, is consistent with the residual activity of the construct 192–221 (Fig. 4A). Surprisingly mutation mut4 improves the balancer activity possibly due to the fact that the 3′ border of the balancer is already partially destroyed in the 39 nt sequence (compare constructs 1–449, 1–249 and 1–221 in Fig. 4A).

To evaluate the significance of the balancer within the context of the long (1–3180) 3′UTR we inserted the mut2 mutation into the full-length construct 1–3180 (Fig. 1). Comparing the activity of the mutated construct to the wild type construct we measured a significantly decreased luciferase activity (Fig. 4C). We conclude that the balancer element is of functional relevance within the entire 3′ UTR of *HNF4A*.

### The *HNF4A* 3′UTR is regulated by miRNAs

**Figure 5:**
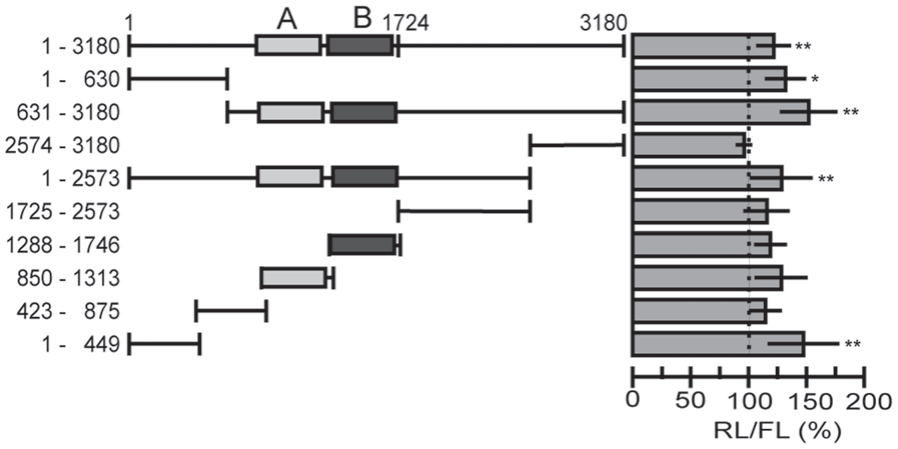
Dicer knock-down indicates that the *HNF4A* 3′UTR is regulated by miRNAs. To knock-down the Dicer protein, doxycycline (1 µg/ml) was added to the Dicer-kd/2b2 cell line [42] for three or seven days. Two days before luciferase activity was measured, the cells were transiently transfected with reporter constructs. The nomenclature of the constructs is as in Fig. 2. At least three transfection assays were performed for each construct, involving at least two independent plasmid preparations. Each assay was performed in triplicate and a CMV-driven firefly luciferase (FL) was used to control for transfection efficiency. The activity of each construct measured in the presence of Dicer (ethanol added) was used for standardization (100%) and is not shown. The 3xBulgeB and RL-Con reporter plasmids [42] harboring three bulged binding sites for let-7a and lacking any binding sites, respectively, were included as a positive and negative control in each experiment (not shown). The negative elements A and B identified in Fig. S1 are indicated. The *p*-values were determined using an independent-samples *t* test. *p*-values of<0.05 and of <0.01 are indicated by * or **, respectively.

To address the question, if *HNF4A* is regulated by miRNA, we searched for binding sites using the RNA22 program [46]. Although we restricted our analysis to 20 miRNAs overexpressed in RCC (Table S3), too many potential binding sites for miRNAs were predicted within the *HNF4A* 3′UTR. Therefore, we evaluated experimentally, if any miRNA targets the 3′UTR of *HNF4A* by using a HEK293 cell line in which the Dicer protein can be conditionally knocked-down by doxycycline [42]. Since Dicer is required for miRNA biogenesis, the repressive effect of miRNAs is relieved, if Dicer is downregulated. Using the *Renilla* luciferase reporter with the entire 3180 nt *HNF4A* 3′UTR, depletion of Dicer resulted in an increase in luciferase reporter activity by 21% (Fig. 5). The effect was not as pronounced as for the artificial reporters RL-Perf containing one perfect let-7a binding site and RL-3xBulgeB containing three bulged let-7a sites [42] that mediated under our conditions an increase of 55% and 89%, respectively. However, the significant increase indicates that miRNAs target the *HNF4A* 3′UTR.

To locate potential miRNA binding sites in the *HNF4A* 3′UTR, we analyzed several fragments of the 3′UTR individually (Fig. 5). Whereas miRNAs did not seem to target the 3′ end of the 3′UTR (2574–3180), the remaining fragments mediated a slight increase in reporter activity upon Dicer knock-down and the 5′ fragment 1–449 nt showed a highly significant effect. In conclusion, our data reveal potential functional miRNA target sites distributed within 2.6 kb of the 3.2 kb 3′UTR.

### *miR-34a* and *miR-21* downregulate *HNF4A*

**Figure 6:**
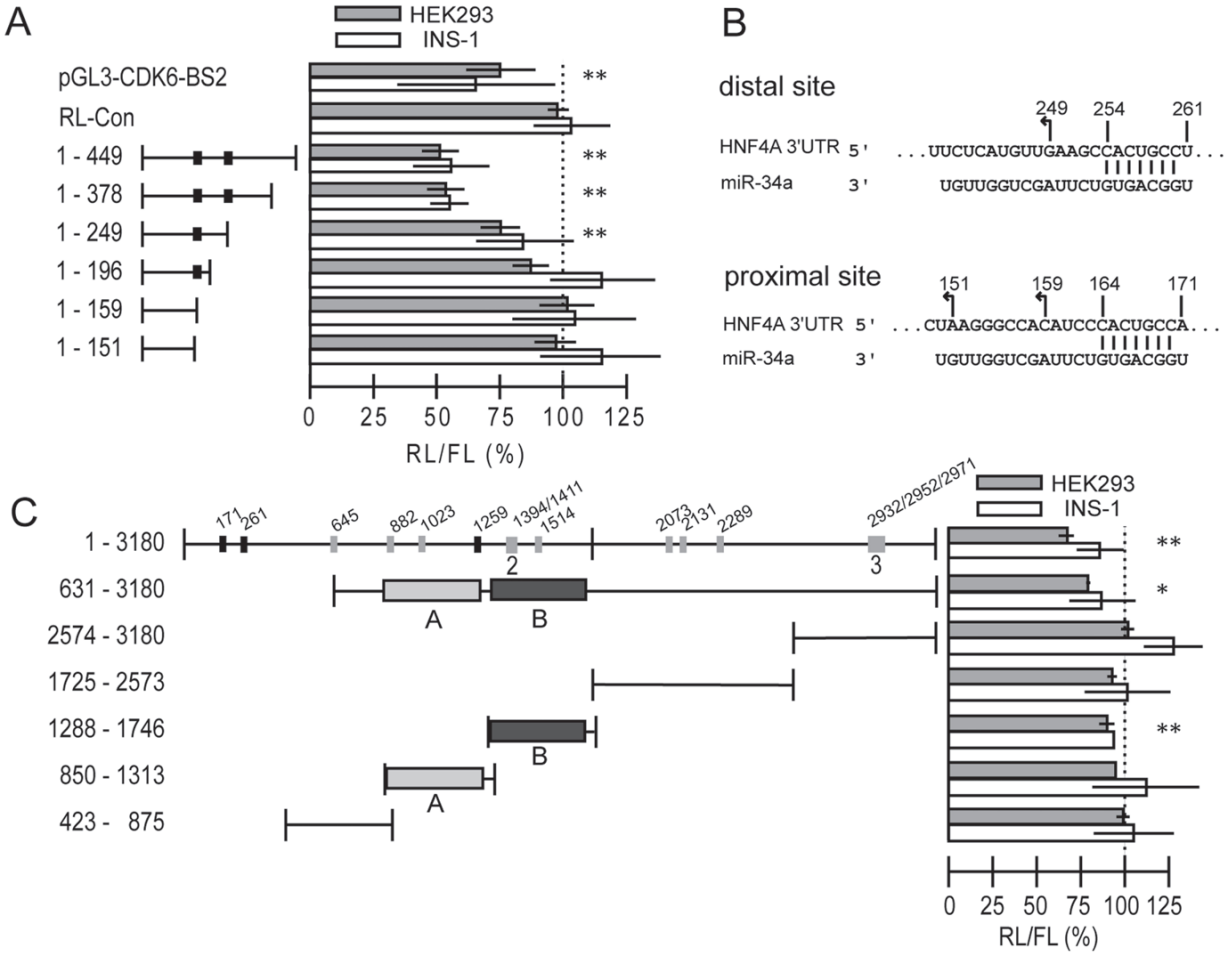
Reporter analyses of miR-34a binding sites in the *HNF4A* 3′UTR. (**A**) *miR-34a* targets two sites within the 5′ 449 nt of the *HNF4A* 3′UTR. Reporter plasmids and *pri-miR-34a* expression plasmids were co-transfected 24 h before cell collection, into HEK293 (upper grey bars) and INS-1 cells (lower white bars). At least one transfection assay was performed for each construct, involving two independent plasmid preparations in the case of two or more assays. Each assay was performed in triplicate and a CMV-driven firefly luciferase was used to control for transfection efficiency. The activity of each construct in the absence of the *pri-miR-34a* plasmid (replaced by Rc/CMV) was used for standardization (100%) and is not shown. pGL3-CDK6-BS2 [44] and pRL-Con [42] served as positive and negative controls, respectively. Since pGL3-CDK6-BS2 expresses the firefly luciferase, the RL-Con plasmid was used to control for transfection efficiency. The black boxes indicate miR-34a target sites with perfect seed sequence. To calculate *p*-values the data of HEK293 and INS-1 cells were combined for each construct. *p*-values are<0.05 (*) and <0.01 (**) using an independent-samples *t* test. (**B**) Schematic diagram of the two potential miR-34a binding sites within the 5′ 449 nt of the *HNF4*A 3′UTR. The numbering refers to the first nucleotide after the stop codon as 1. The site extending from 149–171 nt was only predicted by TargetScan and is little conserved, while the distal site located at 239–261 nt was predicted by RNA22 and TargetScan and is highly conserved among vertebrates. The two perfect seed matches are indicated by vertical lines between the *HNF4A* 3′UTR and miR-34a sequence. The arrows at position 151, 159 and 249 mark the last nucleotide of the *HNF4A* 3′UTR in the corresponding constructs. (**C**) Analyzing the performance of various 3′UTR fragments upon *miR-34a* overexpression as performed in panel A. The grey and black boxes indicate miR-34a target sites without and with a perfect seed sequence **(CACUGCC)**, respectively, as determined by RNA22 [46]. The number of target sites indicated by a box is given underneath the target site in case of more than one site. The nucleotide positions of the 3′ end of the target sites are given. The negative elements A and B identified in Fig. S1 are indicated.

To specify miRNAs that potentially target the *HNF4A* 3′UTR we first selected *miR-34a* that is overexpressed in RCC (Table S3) and contains binding sites with seed sequences within the 5′ 449 nt. Although *miR-34a* is moderately expressed in HEK293 cells (Table S3), overexpression of *pri-miR-34a* downregulated the validated *miR-34a* reporter plasmid pGL3-CDK6-BS2 (Fig. 6A) containing one miR-34a target site [44]. An even more pronounced decrease by *miR-34a* was observed by using reporters including the 5′ end construct 1–449 or 1–378 of the *HNF4A* 3′UTR (Fig. 6A) that contains two potential miR-34a binding sites with perfect seed sequences (Fig. 6B). Deletion of the seed sequence of the distal miR-34a binding site in construct 1–249 clearly diminished the decrease, but did not entirely abrogate the effect (Fig. 6B) suggesting that the proximal site is also functional. Consistent with this assumption constructs 1–159 and 1–151 that both lacked the proximal miR-34a target site were not affected by *miR-34a* (Fig. 6B). Since construct 1–196 that retains the proximal site was also not downregulated by *miR-34a*, we assume that neighboring sequences are also required. Taken together it is evident that the proximal (at 171 nt) and distal (at 261 nt) miR-34a binding site within the 5′ 449 nt are functional and their effect is additive (Fig. 6A and B).

Examination of the entire 3180 nt 3′UTR revealed 13 additional potential miR-34a binding sites (Fig. 6C). Overexpression of *miR-34a* with the long 3′UTR (1–3180) luciferase reporter led to a decreased luciferase activity in HEK293 and INS-1 cells similar to the one measured for pGL3-CDK6-BS2 reporter construct. We ruled out that this decrease was based entirely on the two identified miR-34a binding sites within the 5′ 449 nt fragment, as we measured a *miR-34a* dependent drop in luciferase activity with a construct (631–3180) lacking the 5′ sequence (Fig. 6C). Therefore, we tested several shortened constructs of the *HNF4A* 3′UTR, each containing at least one potential miR-34a binding site. Since the construct 1288–1746 was affected by *miR-34a* overexpression, we assume that this region contains several cooperating miR-34a sites.

**Figure 7:**
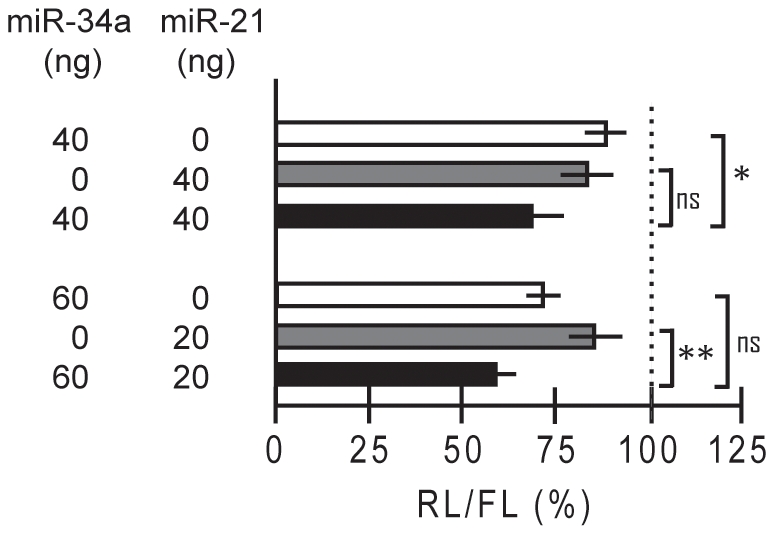
*miR-34a* and *miR-21* cooperate on the *HNF4A* 3′UTR. The reporter plasmid with the full-length *HNF4A* 3′UTR (1–3180) was cotransfected into INS-1 cells with expression vectors encoding *pri-miR-34a* and/or a *miR-21* as indicated. 24 h later *Renilla* luciferase activity was measured and standardized using the cotransfected firefly luciferase reporter. Each experiment involved three assays performed in triplicate and the activity in the presence of the empty expression vector was used for standardization (100%). By adding empty vector the amount of DNA was kept constant. The specificity of the *miR-21* expression vector *pCMV-miR21* [45] was verified by using the bona fide reporter *Luc-TPM1-V1-UTR* and its derivative *Luc-TPM1-V4-UTR* lacking a miR-21 binding site (Fig. S2). *p*-values of >0.05 (ns), <0.05 (*) and <0.01 (**) are calculated as indicated by brackets using an independent-samples *t* test.

In a similar analysis with expression vectors encoding *miR-21* and *miR-122* we observed a regulation of the *HNF4A* 3′UTR by *miR-21* (Fig. S2), but not by *miR-122* (data not shown). Based on *in silico* analysis using RNA22 [46] we identified seven potential *miR-21* binding sites lacking a perfect seed sequence (Fig. S2). Analyzing fragments of the 3′UTR we failed to narrow down the *miR-21* regulation to a specific site (Fig. S2) indicating the cooperation of several sites. Our observation that simultaneous overexpression of *miR-34a* and *miR-21* has an additive effect on the long (1–3180) *HNF4A* 3′UTR (Fig. 7) is most relevant, since both miRNAs are upregulated in RCC (Table S3).

### A SNP in the *HNF4A* 3′UTR affects *miR-34a* function

**Figure 8:**
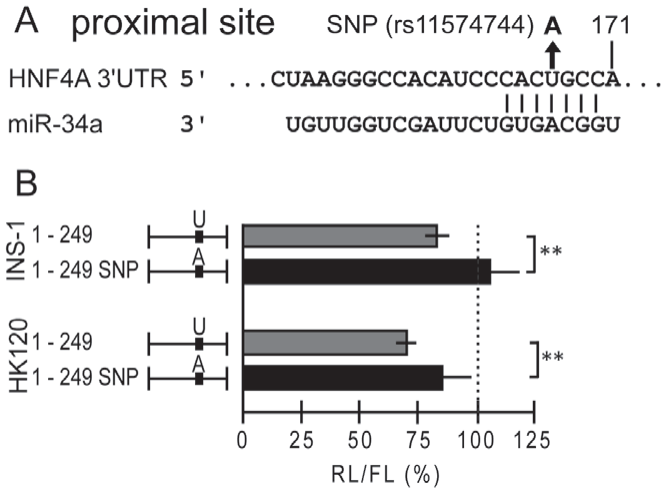
SNP rs11574744 destroys the proximal miR-34a binding site. (**A**) The nucleotide change of the SNP rs11574744 in the proximal miR-34a site 171 nt downstream of the stop codon of the *HNF4A* mRNA is given. (**B**) Luciferase reporter assays were performed in INS-1 and HK120 cells with the expression vector for *miR-34a* and either the *HNF4A* 3′UTR reporter construct 1–249 or the corresponding construct carrying the A nucleotide variant. 100% refers to the activity in the presence of an empty expression vector. ** denotes *p*-values of <0.01 using an independent-samples *t* test. Experimental details are as in Fig. 6.

The *HNF4A* mRNA contains 28 single nucleotide polymorphism (SNP) in the 3180 nt of the long 3′UTR (dbSNP build 130). One of them (rs11574744) alters the U to an A in the seed sequence of the proximal miR-34a site (Fig. 8A). We inserted this nucleotide change into the 1–249 construct that retains the functional proximal miR-34a site (see Fig. 6A). Luciferase reporter assays in INS-1 and HK120 cells revealed the functional relevance of the U nucleotide in the seed sequence, as the SNP fully or partially destroyed the function of the proximal miR-34a site in INS-1 or HK120 cells, respectively (Fig. 8B).

## Discussion

**Figure 9:**
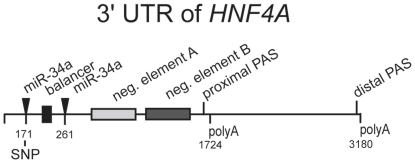
Regulatory elements identified in the 3′UTR of the *HNF4A* mRNA. A summary of the various functional elements identified in our work is illustrated. The SNP rs11574744 located in the proximal miR-34a site (171 nt) is given. The functional relevance of the distal miR-34a site (271 nt) has been reported independently [65]. The numbering starts with the first nucleotide of the 3′UTR after the stop codon.

Gene regulation involves complex networks of *cis*-acting elements and *trans*-acting factors that work on the transcriptional and posttranscriptional level. Whereas on the transcriptional level promoter and enhancer elements with their corresponding DNA binding proteins have been well characterized [49], [50], posttranscriptional control involving the 3′UTR of mRNAs has been largely neglected. This lack of knowledge is quite surprising, as in many cases the 3′UTR of a given mRNA exceeds the length of the ORF substantially [51] as exemplified also by *HNF4A* (Fig. 1). Furthermore, only 39 motifs recognized by RNA binding proteins are deposited in the database UTRdb [47], whereas 457 transcription factor binding sites are available in JASPAR 2010 [52]. In fact, *in silico* analysis of the *HNF4A* 3′UTR using UTRdb [47] reveals the distal PAS and four potential regulatory sites (SXL binding site, position 2680–2695; K-box, position 2940–2947; Musashi binding element at position 910–914 and 2152–2157), none of which explains the functional elements we have identified in this study (Fig. 9).

Investigating the 3′UTR of human *HNF4A* for its regulatory potential we detected a proximal and distal PAS leading to a short (1.7 kb) and long (3.2 kb) 3′UTR, respectively (Fig. 9). This finding is not surprising as about 29% of mRNAs contain more than one PAS [48]. In accordance with data showing that non-canonical signals are processed less efficiently than the canonical PAS [48], the long 3′UTR derived from the canonical PAS is generated predominantly in HepG2 and HK120 cells (Fig. 1B). Although our functional data show that all regulatory elements are included in the short form (Fig. 9), we assume some functional relevance for the long 3′UTR (1–3180) that corresponds to the 3′UTR of the murine *Hnf4a* mRNA.

In a systematic analysis of both 3′UTRs by reporter assays we observed significantly reduced luciferase reporter activity in HEK293 and INS-1 cells, as described for other 3′UTRs in previous studies [53]–[56]. By deletion analysis we identified the negative elements A (850–1207) and B (1288–1666) to confer the highest repressive effect in both cell lines (Fig. 2 and S1). To exclude that transcriptional elements located in the 3′UTR interfere in our assay, we showed that negative elements A and B act on RNA level, as the antisense sequences are not functional and an upstream SV40 transcriptional stop element destroys the repressive function. The size of the negative elements A and B of about 400 nt (Fig. S1) is much larger than a binding site of a RNA binding protein or a miRNA. However, as overexpression of *miR-34a* slightly impairs the activity of fragment 1288–1746 (Fig. 6C) which includes negative element B (1288–1666), a contribution of *miR-34a* cannot be excluded. We assume that the single-stranded RNA containing negative elements A and B adopts a secondary structure as postulated for instance for the 3′UTRs of *Vg1* [57] and *bicoid* [58], [59], that is involved in the cytoplasmic localization of the mRNA.

The pronounced negative effect of element A and B is masked within the *HNF4A* 3′UTRs due to a balancer element located 5′ of these two negative elements (Fig. 9). Although the balancer is flanked by miR-34a binding sites (Fig. 9), it seems unlikely they are relevant for balancer function, as the balancer activity of the construct 1–249 (Fig. 4A) is not altered by the presence of the SNP (data not shown) destroying the miR-34a binding site at 171 (Fig. 8). The balancer acts only in its forward orientation, as expected for a RNA element (Fig. 4A). The 5′ and 3′ borders of the balancer are not very sharp and we define a minimal sequence of 39 nt as the balancer (Fig. 4A). The conserved blocks CTTT and CTTG that are essential for balancer function (Fig. 4B) do not correspond to any known RNA binding site and we found no obvious secondary structure. None of the 28 SNP of the long 3′UTR (1–3180) is located within the balancer (dbSNP build 130). Importantly, we showed that the balancer is also functional within the context of the long 3′UTR of 3180 nucleotides (Fig. 4C). As mutation of only four nucleotides within the 3180 nucleotides of the 3′UTR has a significant effect, sequencing of the 3′UTR in diabetes and RCC patients might be most relevant.

We postulate that regulation of *HNF4A* via its 3′UTR could be modified by an altered interplay of the negative elements A and B with the balancer depending on the level of transacting proteins that potentially target these distinct elements. A similar potential complex regulation mediated by several, distinct functioning 3′UTR elements has also been described in *IGF-II* [60], *Cox-2* [53] and *CDK5R1* [55] mRNAs. However, in all cases a clear biological function is elusive and further examples have not been described up to date.

Since too many potential miRNA targets can be found in the *HNF4A* 3′UTR, we restricted the analysis to 20 miRNAs upregulated in RCC (Table S3) predicting 141 potential miRNA binding sites within the long (1–3180) *HNF4A* 3′UTR. Due to the high false-positive rate of target prediction [61], [62], we performed functional assays. Using a HEK293 cell line with a conditional Dicer knock-down [42] we could locate potential miRNA targets within the 5′ 449 nt of the *HNF4A* 3′UTR (Fig. 5). In accordance with findings describing the effects of miRNAs on proteins as quite modest [63], [64], derepression upon Dicer knock-down was moderate, but significant. In complementary experiments measuring the effect of specific miRNAs we demonstrate the functional relevance of *miR-34a* (Fig. 6) and *miR-21* (Fig. S2). However, our data do not prove an effect on the endogenous *HNF4A* mRNA or HNF4α protein. An experimental link of *miR-34a* and endogenous *HNF4A* mRNA has been verified recently in HepG2 cells where overexpression of *miR-34a* decreased HNF4αprotein [65].

Even though several binding sites without a perfect seed sequence have been proven to be functional, a perfect seed sequence is crucial for miRNA function in most cases [63], [66]. Furthermore, a high number of predicted binding sites for a given miRNA is supposed to facilitate the regulation of a target gene [67]. *miR-34a* fulfilled both criteria and we were able to validate the two bindings sites within the 5′ 449 nt of the *HNF4A* 3′UTR. Both sites contributed equally to repression, a characteristic of independent and non-cooperative action termed multiplicative effect [68]. During preparation of this manuscript *miR-34a* regulation of the *HNF4A* mRNA has been reported independently in HepG2 cells [65]. Our data extend this report that described only the distal miR-34a binding site at 261 nt of the 3′UTR to be involved in translational repression of *HNF4A*. In addition, we also verified the *miR-34a* dependent repression of the long 3180 nt *HNF4A* 3′UTR (Fig. 6C) and by applying our assay in a renal and pancreatic cell type we established *miR-34a* function in distinct cofactor environments. Since the remaining 13 potential miR-34a binding sites were functioning within construct 631–3180 and three even in construct 1288–1746 (Fig. 6C), we deduce multiple control elements in the 3′UTR of *HNF4A* that are targeted by *miR-34a*. Furthermore, we establish the significance of a single nucleotide exchange in the CACUGCC seed sequence of the *miR-34a* target site [69], as SNP rs11574744 in the seed sequence of the proximal miR-34a target site at 171 nt destroys the function (Fig. 8). This SNP contains an A instead of a T and has an allele frequency of 0.020–0.025 (http://www.ncbi.nlm.nih.gov/projects/SNP/), but so far no phenotype has been associated with this SNP. Since potentially 15 miR-34a sites are present in the *HNF4A* 3′UTR (Fig. 6C), the destruction of a single site may have a minimal effect, unless each miR-34a site plays its unique role.

*miR-34a* has primarily been characterized as a tumor suppressor, as it is inactivated in several tumors and transcriptionally activated by p53. In addition, ectopic *miR-34a* expression induces apoptosis, cell cycle arrest or senescence [69], [70]. However, *miR-34a* is upregulated in RCC (Table S3), hepatocellular carcinoma [71], breast cancer [72], squamous cell lung carcinoma [73] and in chronic lymphocytic leukemia [74]. Thus, it appears that *miR-34a* acts as a tumor suppressor or an oncogene, depending on the cell type specific targets and regulatory mechanisms. This observation has been established for several other miRNAs [75]. In fact, *miR-34a* was clearly increased in stress induced renal carcinogenesis of the rat and inhibition of *miR-34a* significantly decreased cell proliferation in a rat RCC cell line, but also in HeLa and MCF-7 cells [34]. Furthermore, the oncogenic potential of *miR-34a* was implied by its upregulation in RCC and the correlated decrease of the tumor suppressor SFRP1 whose loss has been observed in a majority of RCC [76].

Our data also imply a regulation of the *HNF4A* 3′UTR by *miR-21* (7), but we were unable to locate a specific target site (Fig. S2). We assume that the regulation involves the cooperation of several sites predicted *in silico* (Table S3). Since *miR-21* is overexpressed in many tumors [77], including RCC (Table S3), we propose that the cooperation of *miR-34a* and *miR-21* in the reporter assays (Fig. 7) may also be operating in tumorigenesis. Therefore, we speculate that the concerted upregulation of *miR-34a* and *miR-21* potentially causes the downregulation of *HNF4A* in RCC, resulting in increased cell proliferation through misregulation of at least 15 HNF4αtarget genes involved in proliferation control [5]. As loss of *HNF4A* is linked to type II diabetes [7], [8] and plays a crucial role in MODY1 [9], [10], it is most relevant to learn whether *miR-34a* and *miR-21* are dysregulated in pancreatic β-cells of patients. Interestingly, for *miR-34a* an increased serum level has been reported in type 2 diabetes patients [78], but it remains open whether this reflects a corresponding increase in β-cells.

In conclusion, our experiments show that *HNF4A* is not only regulated on the transcriptional level via its P1 [79] and P2 [20] promoters and the enhancer [79], but also on the posttranscriptional level via several distinct elements in the two 3′UTRs (Fig. 9).

## Supporting Information

Figure S1 (PDF)

Figure S2 (PDF)

Table S1 (PDF)

Table S2 (PDF)

Table S3 (PDF)
