# Supplementary material for: A Systematic Analysis of the 3′UTR of HNF4A mRNA Reveals an Interplay of Regulatory Elements Including miRNA Target Sites
Source: PLoS One. 2011 Nov 30;6(11):e27438. doi: 10.1371/journal.pone.0027438 (PMC3227676; doi:10.1371/journal.pone.0027438)
Supplement: Figure S1 — (PDF) [file pone.0027438.s001.pdf]

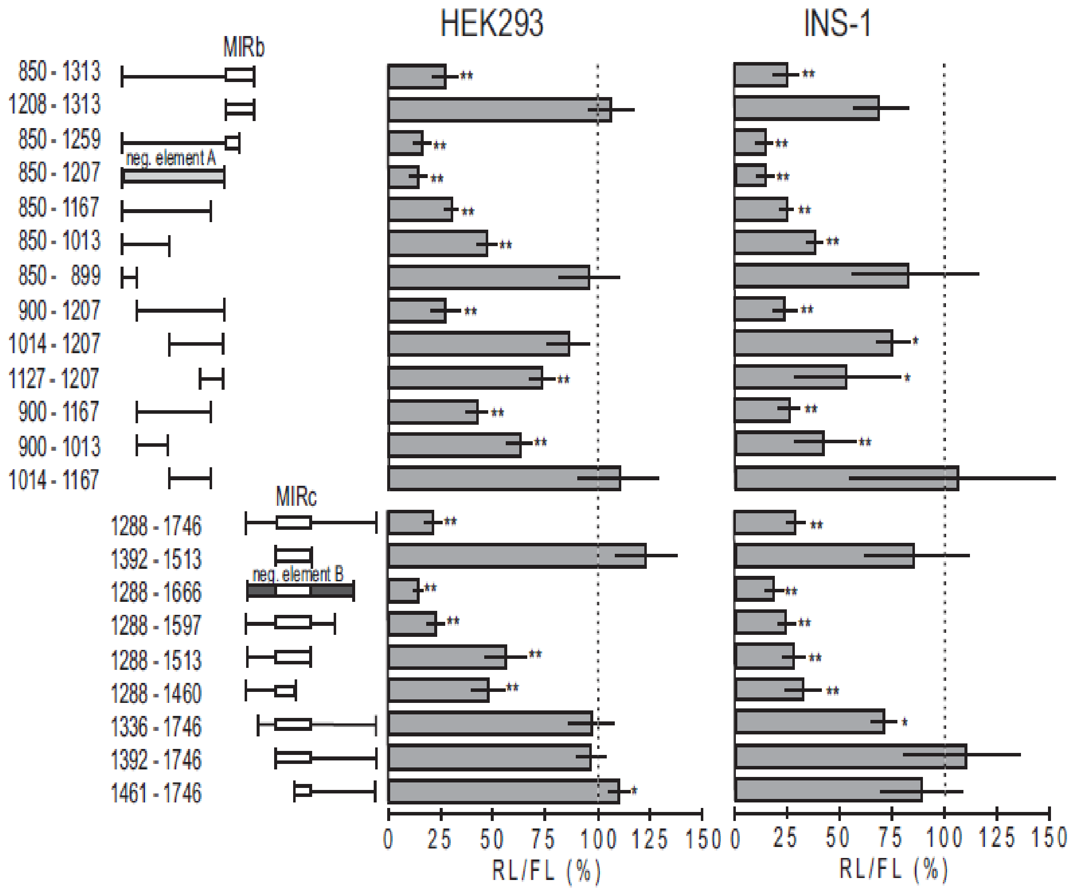


**Figure S1** Locating negative elements A and B in the *HNF4A* 3'UTR. The results of luciferase assays were derived and evaluated as in Fig 2.
