# Supplementary material for: A Systematic Analysis of the 3′UTR of HNF4A mRNA Reveals an Interplay of Regulatory Elements Including miRNA Target Sites
Source: PLoS One. 2011 Nov 30;6(11):e27438. doi: 10.1371/journal.pone.0027438 (PMC3227676; doi:10.1371/journal.pone.0027438)
Supplement: Figure S2 — (PDF) [file pone.0027438.s002.pdf]

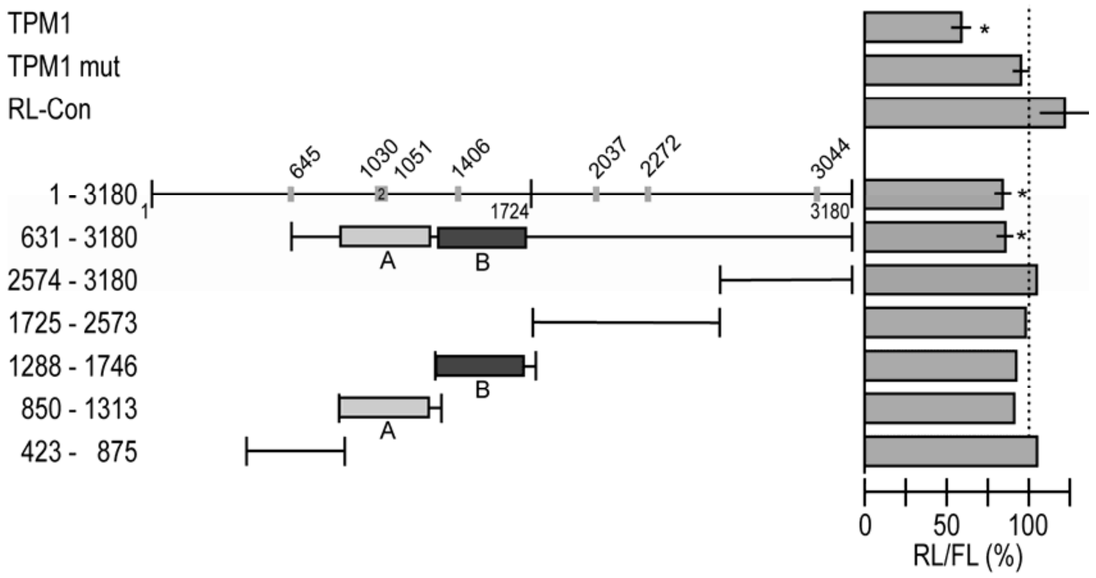

Figure S2 Reporter analyses of miR-21 binding sites in the *HNF4A* 3'UTR. HEK293 cells were co-transfected with reporter plasmids and miRNA expression vectors pCMV-miR-21 [1] 24 h before cell collection. At least one transfection assay was performed for each construct, involving two independent plasmid preparations in case of two or more assays. Each assay was performed in triplicate and a CMV-driven *firefly* luciferase was used to control for transfection efficiency. The activity of each construct in the absence of the miRNA expression plasmid (replaced by Rc/CMV) was used for standardization (100%) and is not shown. TPM1 (Luc-TPM1-V1-UTR) [1] served as positive, while TPM1 mut (Luc-TPM1-V1-UTR-d) [1] was used as a negative control for *miR-21*. The grey boxes indicate potential miRNA target sites without a perfect seed sequence and the number of target sites is given within the box in case of more than one site. The 3' end of the miR-21 target site identified by RNA22 [2] is given. *p*-values were determined using a one-sample *t* test. *p* values of < 0.05 are indicated by *. The negative elements A and B identified in Fig. S1 are indicated.

Reference List

1. Zhu S, Si ML, Wu H, Mo YY (2007) MicroRNA-21 targets the tumor suppressor gene tropomyosin 1 (TPM1). J Biol Chem 282: 14328-14336.

2. Miranda KC, Huynh T, Tay Y, Ang YS, Tam WL et al. (2006) A pattern-based method for the identification of MicroRNA binding sites and their corresponding heteroduplexes. Cell 126: 1203-1217.
