# Supplementary material for: A Systematic Analysis of the 3′UTR of HNF4A mRNA Reveals an Interplay of Regulatory Elements Including miRNA Target Sites
Source: PLoS One. 2011 Nov 30;6(11):e27438. doi: 10.1371/journal.pone.0027438 (PMC3227676; doi:10.1371/journal.pone.0027438)
Supplement: Table S1 — (PDF) [file pone.0027438.s003.pdf]

Table S1: Primers used to amplify the *HNF4A* 3'UTR.

Primers (5'-3') for the *HNF4A* 3'UTR (nt) constructs

1 - 3180	GG**ACTAGT**TAGCAAGCCGCTGGG
	GCATGCGGCCGCTTAGAAAACATATGCGCCATTT
1 - 2769	GG**ACTAGT**TAGCAAGCCGCTGGG
	GCATGCGGCCGCTGTCCCCCCAGCAAC
1 - 2573	GG**ACTAGT**TAGCAAGCCGCTGGG
	GCATGCGGCCGCCCTCCAGAAAGGGGTAGATTC
1 - 1746	GG**ACTAGT**TAGCAAGCCGCTGGG
	GCATGCGGCCGCGAGAAAAGCTGTCAAGAGTCATGA
1 - 630	GG**ACTAGT**TAGCAAGCCGCTGGG
	GCATGCGGCCGCCCCTGCCTGGTGCCT
1 - 449	GG**ACTAGT**TAGCAAGCCGCTGGG
	GCATGCCGGCCGCTGCCCCAAGTGCCAC
1 - 378	GG**ACTAGT**TAGCAAGCCGCTGGG
	GCATGCGGCCGCGAGAAGCACCAGGCTAGGG
1 - 249	GG**ACTAGT**TAGCAAGCCGCTGGG
	GCATGCGGCCGCCAACATGAGAAAAGTTGTCCAAG
1 - 196	GG**ACTAGT**TAGCAAGCCGCTGGG
	GCATGCGGCCGCGTTATCCAGAGCAGGGCGT
1 - 159	GG**ACTAGT**TAGCAAGCCGCTGGG
	GCATGCGGCCGCGTGGCCCTTAGGCCATG
1 - 151	GG**ACTAGT**TAGCAAGCCGCTGGG
	GCATGCGGCCGCTAGGCCATGTTCTCGGG
423 - 875	GG**ACTAGT**CTGGGTCCAATTGTGGCA
	GCATGCGGCCGCTCCCATCTCACCTGCTCTACC
850 - 899	GC***TCTAGA***TGGCTGGTAGAGCAGGTGA
	GCATGCGGCCGCTGGCTCAGGCTGTTCTTTG
850 - 1013	GC***TCTAGA***TGGCTGGTAGAGCAGGTGA
	GCATGCGGCCGCCTCAGCCTGGTGTTCCAGA
850 - 1167	GC***TCTAGA***TGGCTGGTAGAGCAGGTGA
	GCATGCGGCCGCGTCCTCTCCAGCCCCAAG
850 - 1207	GC***TCTAGA***TGGCTGGTAGAGCAGGTGA
	GCATGCGGCCGCCCTCCTGATGTCACTCTGAT
850 - 1259	GC***TCTAGA***TGGCTGGTAGAGCAGGTGA
	GCATGCGGCCGCAGACAGTGCCTGGGAGTAAGG
850 - 1313	GG**ACTAGT**TGGCTGGTAGAGCAGGTGA
	GCATGCGGCCGCGGTTAATAGGGAGGAAGGGAGG
900 - 1013	GC***TCTAGA***AGGCCTAGTGGTAGTAAGAATCTAGC
	GCATGCGGCCGCCTCAGCCTGGTGTTCCAGA
900 - 1167	GC***TCTAGA***AGGCCTAGTGGTAGTAAGAATCTAGC
	GCATGCGGCCGCGTCCTCTCCAGCCCCAAG
900 - 1207	GC***TCTAGA***AGGCCTAGTGGTAGTAAGAATCTAGC
	GCATGCGGCCGCCCTCCTGATGTCACTCTGAT
1014 - 1167	GC***TCTAGA***GTCCTGATCAGCTTCAAGGAGT
	GCATGCGGCCGCGTCCTCTCCAGCCCCAAG
1014 - 1207	GC***TCTAGA***GTCCTGATCAGCTTCAAGGAGT

	GCATGCGGCCGCCCTCCTGATGTCACTCTGAT
1127 - 1207	GC***TCTAGA***TAATGCGGGTGAGAGTAATGAG
	GCATGCGGCCGCCCTCCTGATGTCACTCTGAT
1208 - 1313	GC***TCTAGA***AATAAGCTCCCAGGGCCTG
	GCATGCGGCCGCGGTTAATAGGGAGGAAGGGAGG
1288 - 1460	GC***TCTAGA***TAATCCTCCCTTCCTCCCTATT
	GCATGCGGCCGCCTTCCTAGTTGTGTGAGTTTCAGAA
1288 - 1513	GC***TCTAGA***TAATCCTCCCTTCCTCCCTATT
	GCATGCGGCCGCAAGAGCTCCTGTTCTGATCCAG
1288 - 1597	GC***TCTAGA***TAATCCTCCCTTCCTCCCTATT
	GCATGCGGCCGCTGTAGAAGGGAGCCGGAAG
1288 - 1666	GC***TCTAGA***TAATCCTCCCTTCCTCCCTATT
	GCATGCGGCCGCCAGCCTCAGGCCAATCTT
1288 - 1746	GG**ACTAGT**TAATCCTCCCTTCCTCCCTATT
	GCATGCGGCCGCGAGAAAAGCTGTCAAGAGTCATGA
1336 - 1746	GC***TCTAGA***TTCTCCTCCTCCCTCCCC
	GCATGCGGCCGCGAGAAAAGCTGTCAAGAGTCATGA
1392 - 1513	GC***TCTAGA***TTACAGAAGCTGAAATTGCGTTC
	GCATGCGGCCGCAAGAGCTCCTGTTCTGATCCAG
1392 - 1746	GC***TCTAGA***TTACAGAAGCTGAAATTGCGTTC
	GCATGCGGCCGCGAGAAAAGCTGTCAAGAGTCATGA
1461 - 1746	GC***TCTAGA***TGGCTGAGTCAGGACTTGAA
	GCATGCGGCCGCGAGAAAAGCTGTCAAGAGTCATGA
1725 - 2573	GG**ACTAGT**ATGACTCTTGACAGCTTTTCTCTCT
	GCATGCGGCCGCCCTCCAGAAAGGGGTAGATTC
2574 - 3180	GG**ACTAGT**AGAAACCCATTCCACCTTAATAAC
	GCATGCGGCCGCTTAGAAAACATATGCGCCATTT
2771 - 3180	GG**ACTAGT**AGCGTGGGCACAATTTC
	GCATGCGGCCGCTTAGAAAACATATGCGCCATTT

The forward primers used to amplify parts of the *HNF4A* 3'UTR are always listed first and the reverse primers second. The forward primers are either flanked by a *Spe*I (bold) or *Xba*I (bold and italics) restriction site, while the reverse primers contain a *Not*I (underlined) site for ligation into the *Xba*I/*Not*I sites downstream of the *Renilla* luciferase into the RL-Con plasmid. The numbers refer to the position in the 3180 nt of the 3'UTR starting with the first nucleotide after the stop codon (see Fig. 1A).

Primers to clone the sequence containing the SV40 PAS

GC***TCTAGA***TTCCCTTTAGTGAGGGTTAATGC
GG**ACTAGT**ATCACCCTAATCAAGTTTTTGGG

The primers are either flanked by a *Spe*I (bold) or *Xba*I (bold and italics) restriction site.

Primers to define the balancer

1 – 449	GG**ACTAGT**TAGCAAGCCGCTGGG
	GC***TCTAGA***TGCCCCAAGTGCCAC
1 – 249	GG**ACTAGT**TAGCAAGCCGCTGGG
	GC***TCTAGA***CAACATGAGAAAAGTTGTCCAAG
1 – 233	GG**ACTAGT**TAGCAAGCCGCTGGG
	GC***TCTAGA***GTCCAAGGCAGTAGAGG
1 – 221	GG**ACTAGT**TAGCAAGCCGCTGGG
	GC***TCTAGA***GAGGTCTCCCCAAGTCAAAG
1 – 214	GG**ACTAGT**CAAGCCGCTGGGGGC
	GC***TCTAGA***TCCCCAAGTCAAAGTCTTG
1 – 210	GG**ACTAGT**CAAGCCGCTGGGGGC
	GC***TCTAGA***CAAGTCAAAGTCTTGTTATCCAG
1 – 204	GG**ACTAGT**CAAGCCGCTGGGGGC
	GC***TCTAGA***AAGTCTTGTTATCCAGAGCAG
1 – 196	GG**ACTAGT**CAAGCCGCTGGGGGC
	GC***TCTAGA***GTTATCCAGAGCAGGGCGT
45 -221	GG**ACTAGT**CTAAGAGAGCACCTGGTG
	GC***TCTAGA***GAGGTCTCCCCAAGTCAAAG
86 – 221	GG**ACTAGT**GACGTGATGCCAGGACC
	GC***TCTAGA***GAGGTCTCCCCAAGTCAAAG
132 – 221	GG**ACTAGT**GGGCCCGAGAACATGG
	GC***TCTAGA***GAGGTCTCCCCAAGTCAAAG
170 – 221	GG**ACTAGT**CACCCTTGACGCCCTG
	GC***TCTAGA***GAGGTCTCCCCAAGTCAAAG
178 – 221	GG**ACTAGT**ACGCCCTGCTCTGGATAAC
	GC***TCTAGA***GAGGTCTCCCCAAGTCAAAG
183 – 221	GG**ACTAGT**CTGCTCTGGATAACAAGACTTTG
	GC***TCTAGA***GAGGTCTCCCCAAGTCAAAG
192 – 221	GG**ACTAGT**ATAACAAGACTTTGACTTGGGG
	GC***TCTAGA***GAGGTCTCCCCAAGTCAAAG

The primers are flanked by a *Spe*I (bold) or *Xba*I (bold and italics) restriction site and cloned into the *XbaI* site of the construct containing the negative element A downstream of the *Renilla* luciferase of pRL-Con.
