# Supplementary material for: A Systematic Analysis of the 3′UTR of HNF4A mRNA Reveals an Interplay of Regulatory Elements Including miRNA Target Sites
Source: PLoS One. 2011 Nov 30;6(11):e27438. doi: 10.1371/journal.pone.0027438 (PMC3227676; doi:10.1371/journal.pone.0027438)
Supplement: Table S2 — (PDF) [file pone.0027438.s004.pdf]

Table S2: Oligonucleotides to define the balancer

Polylinker oligonucleotides

5′-CTAG**GAATTC**GTCGACCCCGGG**CTCGAG**
CTTAAGCAGCTGGGGCCC**GAGCTC**GACT-5′

The *Eco*RI (GAATTC) and *Xho*I site (CTCGAG) are given.

Balancer oligonucleotides (183 – 221)

wild type

AATTCTGCTCTGGATAACAAGACTTTGACTTGGGGAGACCTC
GACGAGACCTATTGTTCTGAAACTGAACCCCTCTGGAGAGCT

mut1

AATTCTGC**AGACC**ATAACAAGACTTTGACTTGGGGAGACCTC
GACG**TCTGG**TATTGTTCTGAAACTGAACCCCTCTGGAGAGCT

mut2

AATTCTGCTCTGGATAACAAGA**GAAA**GACTTGGGGAGACCTC
GACGAGACCTATTGTTCT**CTTT**CTGAACCCCTCTGGAGAGCT

mut3

AATTCTGCTCTGGATAACAAGACTTTGA**GAAC**GGGAGACCTC
GACGAGACCTATTGTTCTGAAACT**CTTG**CCCTCTGGAGAGCT

mut4

AATTCTGCTCTGGATAACAAGACTTTGACTTGGGG**TCTG**CTC
GACGAGACCTATTGTTCTGAAACTGAACCCC**AGAC**GAGAGCT

Oligonucleotides with *Eco*RI and *Xho*I overhangs are given. The mutated nucleotides are underlined in the wild type sequence and are in bold letters in the mutated (mut) oligonucleotides.
