# Supplementary material for: A Systematic Analysis of the 3′UTR of HNF4A mRNA Reveals an Interplay of Regulatory Elements Including miRNA Target Sites
Source: PLoS One. 2011 Nov 30;6(11):e27438. doi: 10.1371/journal.pone.0027438 (PMC3227676; doi:10.1371/journal.pone.0027438)
Supplement: Table S3 — (PDF) [file pone.0027438.s005.pdf]

Table S3: Set of miRNAs upregulated in RCC.

miRNA	References	Fold induction	N of target sites (seed)	CT
**miR-7**	1	1.25	8 (1)	33.4
**miR-18a***	2, 6	4.05	12 (1)	33.1 (miR-18a)
**miR-21**	2, 4, 5, 6	3.50	7 (0)	34.3
**miR-27a**	4	1.85	13 (1)	35.3
**miR-34a**	2, 5, 6	2.95	15 (3)	34.2
**miR-106b***	4	2.30	9 (2)	29.8 (miR-106b)
**miR-122**	2, 3, 4, 6	28.16	9 (0)	> 40
**miR-140-5p**	4	10.63	4 (2)	31.7
**miR-146b**	2	1.70	2 (1)	35.0
**miR-155**	2, 3, 5, 6	5.66	2 (0)	36.6
**miR-193a-3p**	6	2.20	1 (1)	35.6 (miR-193a-5p)
**miR-210**	2, 3, 4, 5, 6	11.85	9 (0)	33.1
**miR-224**	2, 3, 4, 5, 6	7.05	2 (0)	> 40
**miR-340***	4	2.41	9 (1)	34.1 (miR-340)
**miR-342-3p**	4	2.35	4 (1)	29.5
**miR-342-5p**	2	2.30	10 (1)	29.5 (miR-342-3p)
**miR-452***	2, 3, 6	8.50	2 (1)	> 40 (miR-452)
**miR-584**	4	2.91	14 (1)	n.d.
**miR-592**	5	4.95	4 (1)	n.d.
**miR-1271**	4	3.86	5 (1)	n.d.

miRNAs are listed, if reported at least in one of the six studies to be upregulated in RCC [1–6] and having a perfect seed sequence within the 3180 nt of the *HNF4A* 3'UTR or if identified in more than four of six studies with a potential target site lacking a perfect seed sequence. For each miRNA the studies reporting an increase are given. We thank Eric J. Kort for providing his original data on miRNA expression profiling [2]. miRNA target analysis was made with RNA22 [7]. For miR-34a an additional site was identified by TargetScan. The predicted number of target sites including perfect seed sequences is given with the perfect seed sites also listed in brackets. The average fold change for a miRNA is used when identified in more than one study. The CT values were determined in HEK293 cells by qRT-PCR using 384 TaqMan human miRNA assays from Applied Biosystems (see Materials and Methods). In cases only the opposite stem loop of the corresponding miRNA (see http://www.mirbase.org/) was on the TaqMan Array this is indicated. miRNAs not analyzed are marked as not determined (n.d.).

Reference List

1. Gottardo F, Liu CG, Ferracin M, Calin GA, Fassan M et al. (2007) Micro-RNA profiling in kidney and bladder cancers. Urol Oncol 25: 387-392.

2. Kort EJ, Farber L, Tretiakova M, Petillo D, Furge KA et al. (2008) The E2F3-Oncomir-1 axis is activated in Wilms' tumor. Cancer Res 68: 4034-4038.

3. Nakada C, Matsuura K, Tsukamoto Y, Tanigawa M, Yoshimoto T et al. (2008) Genome-wide microRNA expression profiling in renal cell carcinoma: significant down-regulation of miR-141 and miR-200c. J Pathol 216: 418-427.

4. Chow TF, Youssef YM, Lianidou E, Romaschin AD, Honey RJ et al. (2009) Differential expression profiling of microRNAs and their potential involvement in renal cell carcinoma pathogenesis. Clin Biochem 43: 150-158.

5. Juan D, Alexe G, Antes T, Liu H, Madabhushi A et al. (2009) Identification of a MicroRNA Panel for Clear-cell Kidney Cancer. Urology 75: 835-841.

6. Jung M, Mollenkopf HJ, Grimm C, Wagner I, Albrecht M et al. (2009) MicroRNA profiling of clear cell renal cell cancer identifies a robust signature to define renal malignancy. J Cell Mol Med 13: 3918-3928.

7. Miranda KC, Huynh T, Tay Y, Ang YS, Tam WL et al. (2006) A pattern-based method for the identification of MicroRNA binding sites and their corresponding heteroduplexes. Cell 126: 1203-1217.
